# The neuropathological landscape of Hispanic and non-Hispanic White decedents with Alzheimer disease

**DOI:** 10.1186/s40478-023-01574-1

**Published:** 2023-06-29

**Authors:** Rebeca Scalco, Naomi Saito, Laurel Beckett, My-Le Nguyen, Emily Huie, Hsin-Pei Wang, Delaney A. Flaherty, Lawrence S. Honig, Charles DeCarli, Robert A. Rissman, Andrew F. Teich, Lee-Way Jin, Brittany N. Dugger

**Affiliations:** 1 Department of Pathology and Laboratory Medicine, School of Medicine, University of California Davis, 4645 2Nd Ave, 3400A Research Building III, Sacramento, CA 95817 USA; 2 Division of Biostatistics, Department of Public Health Sciences, University of California Davis, Davis, CA USA; 3 Taub Institute for Research On Alzheimer’s Disease and Aging Brain, Department of Pathology and Cell Biology, Columbia University Medical Center, New York, NY USA; 4 Taub Institute for Research On Alzheimer’s Disease and Aging Brain, Department of Neurology, Columbia University Medical Center, New York, NY USA; 5 Alzheimer’s Disease Research Center, Department of Neurology, School of Medicine, University of California Davis, Sacramento, CA USA; 6 Department of Neurosciences, University of California San Diego, La Jolla, San Diego, CA USA

**Keywords:** Autopsy, Neurodegeneration, Tauopathy, Latino, LatinX, Disparities, Dementia, Alzheimer disease research centers, Histology

## Abstract

Despite the increasing demographic diversity of the United States’ aging population, there remain significant gaps in post-mortem research investigating the ethnoracial heterogeneity in the neuropathological landscape of Alzheimer Disease (AD). Most autopsy-based studies have focused on cohorts of non-Hispanic White decedents (NHWD), with few studies including Hispanic decedents (HD). We aimed to characterize the neuropathologic landscape of AD in NHWD (*n* = 185) and HD (*n *= 92) evaluated in research programs across three institutions: University of California San Diego, University of California Davis, and Columbia University. Only persons with a neuropathologic diagnosis of intermediate/high AD determined by NIA Reagan and/or NIA-AA criteria were included. A frequency-balanced random sample without replacement was drawn from the NHWD group using a 2:1 age and sex matching scheme with HD. Four brain areas were evaluated: posterior hippocampus, frontal, temporal, and parietal cortices. Sections were stained with antibodies against Aβ (4G8) and phosphorylated tau (AT8). We compared the distribution and semi-quantitative densities for neurofibrillary tangles (NFTs), neuropil threads, core, diffuse, and neuritic plaques. All evaluations were conducted by an expert blinded to demographics and group status. Wilcoxon’s two-sample test revealed higher levels of neuritic plaques in the frontal cortex (*p* = 0.02) and neuropil threads (*p* = 0.02) in HD, and higher levels of cored plaques in the temporal cortex in NHWD (*p* = 0.02). Results from ordinal logistic regression controlling for age, sex, and site of origin were similar. In other evaluated brain regions, semi-quantitative scores of plaques, tangles, and threads did not differ statistically between groups. Our results demonstrate HD may be disproportionately burdened by AD-related pathologies in select anatomic regions, particularly tau deposits. Further research is warranted to understand the contributions of demographic, genetic, and environmental factors to heterogeneous pathological presentations.

**Supplementary Information:**

The online version contains supplementary material available at 10.1186/s40478-023-01574-1.

## Introduction

Alzheimer Disease (AD) is the most prevalent neurodegenerative brain disease and the leading cause of dementia globally [18, 40]. AD is often associated with other pathological changes and can cause irreversible damage to neurons, cell death, and brain atrophy, resulting in progressive cognitive deterioration [40]. The number of individuals in the United States aged 65 years and older is more than 55 million (16.8% of the total population), and around one out of eight Americans in this group is afflicted by AD. This number is projected to double by 2050 [9, 17]. Moreover, socioeconomic disparities within the United States population significantly impact the access to diagnosis, care, and treatment for demented persons, especially since AD and related disorders (ADRDs) disproportionally affect individuals from historically excluded ethnic groups. [6, 12, 26, 35, 46, 73]. Considering this exponential growth, ADRDs are emerging as the most significant challenge for healthcare systems worldwide. Since 2012, the World Health Organization has declared dementia a public health priority in an effort to raise awareness and mobilize collective international action from governments and policy-makers [60]. Further, in the same year, the National Alzheimer's Project Act was published in the United States with the objective of reducing dementia disparities and developing effective strategies for prevention and care for all individuals [23, 41].

When discussing AD disparities, there is a need to expand the current knowledge especially in post-mortem studies on underrepresented persons of certain race and/or ethnic groups [57]. Race and ethnicity are terms ubiquitously used in the medical literature, often interchangeably, despite their fundamentally differing definitions. Historically, race has been used to define persons with a common ancestral background and/or similar phenotypic traits, while ethnicity refers to an individual’s cultural identity and traditions. It is important to note these terms are social constructs, with many aspects influencing differences reported in literature, including but not limited to access to care, education, poverty, living conditions, culture, stress, and systemic, institutional, and individual racism [11, 78, 79]. The Hispanic population is the largest and fastest-growing ethnic group in the US, reaching 62.1 million in 2020 and accounting for 19% of the nation's total population [17]. There is evidence demographic, genetic, and/or environmental differences can result in distinct risks and manifestations of AD among different ethnic groups [19, 28, 30, 34]. Epidemiological studies have shown persons self-identifying as Hispanic are 1 to 1.5 times more likely to be diagnosed with AD and may also exhibit the onset of dementia symptoms earlier in life when compared to non-Hispanic White individuals [9, 62, 70, 73]. According to the Alzheimer’s Association Facts and Figures report in 2023, approximately 12 to 14% of Hispanic individuals who are 65 or older have been diagnosed with ADRDs in the United States, although the cause for the increased prevalence remain poorly elucidated [9, 44, 62]. Therefore, a comprehensive understanding of the ethnoracial determinants of health, particularly those hypothesized to influence the pathogenesis of ADRDs, is instrumental to mitigate risk factors and aid in early recognition of the disease process.

The definitive diagnosis of the underlying causes of AD can only be established through histopathological evaluation of the brain at autopsy [21, 67]. The neuropathological hallmarks of AD feature extracellular aggregated amyloid β (Aβ) protein in the form of Aβ plaques and intraneuronal aggregated hyperphosphorylated tau protein in the form of neurofibrillary tangles (NFTs) and neuropil threads [21]. Despite the increasing demographic diversity of the United States population, there remain significant gaps in postmortem research investigating the ethnoracial heterogeneity in the neuropathological landscape of ADRDs [9, 16, 31, 38, 57]. Most autopsy-based studies, including the frequently used neuropathologic scales (BrainNet Europe, Thal, Braak, CERAD), have been conducted almost exclusively on brains of individuals of White European ancestries, with very few studies involving individuals from other ethnic groups, particularly Hispanic decedents [4, 15, 26, 31, 38, 50, 57, 64, 69, 72, 76]. Here, our objective was to characterize the neuropathologic landscape of AD, denoting the distribution and densities of hallmark AD pathologies – NFTs, neuropil threads (NT’s) and plaques (diffuse, cored, and neuritic) in persons of Hispanic descent. To accomplish this, we utilized brain tissues from Hispanic and non-Hispanic White decedents across three research programs that encompassed Alzheimer’s Disease Research Centers at Columbia University, University of California San Diego, and University of California Davis.

## Material and methods

### Cohort selection

**Fig. 1:**
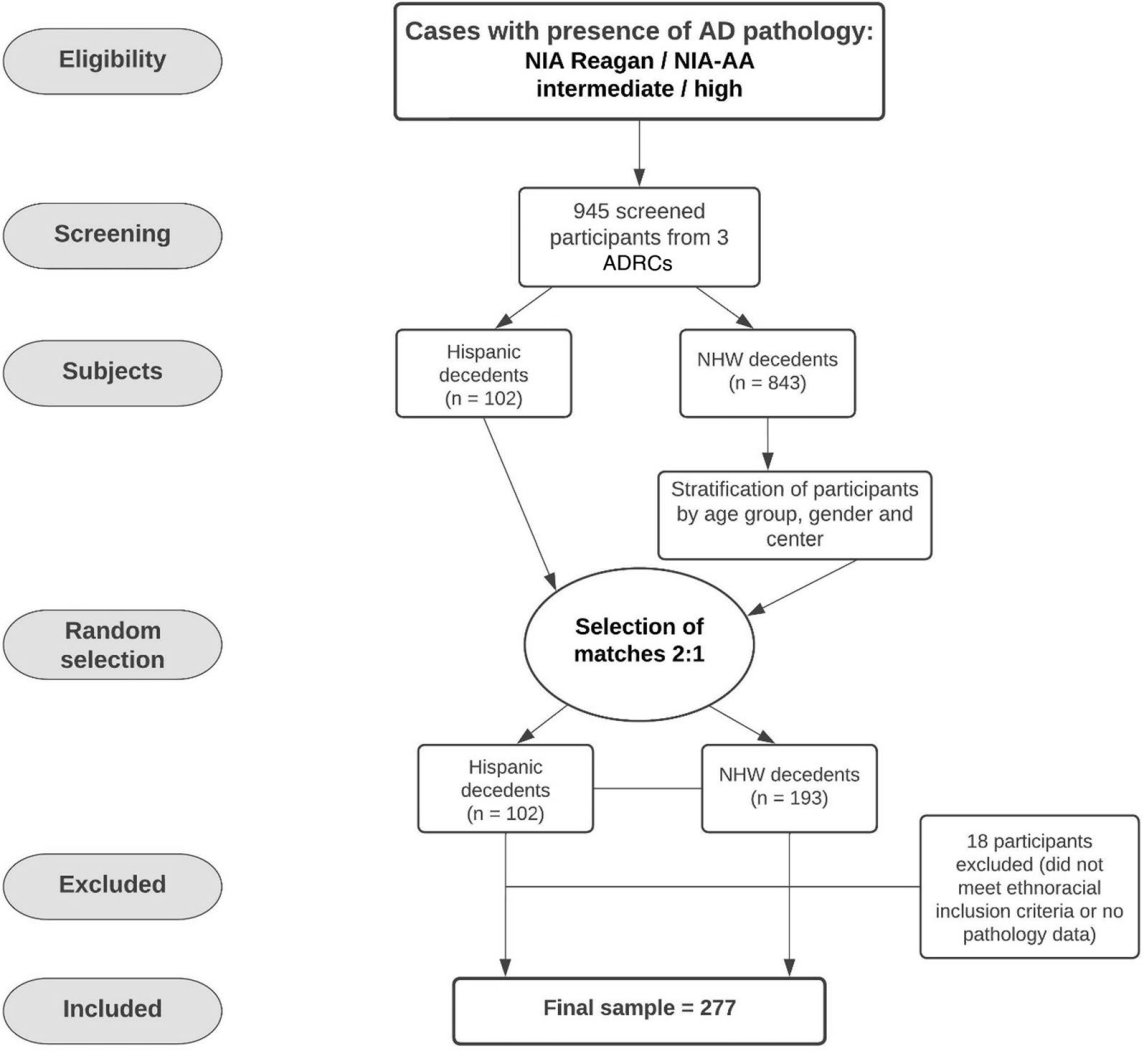
Participant flow diagram summarizing cohort selection, screening, random selection and matching of cases, and inclusion and exclusion criteria in the study

In our study, autopsy brain tissue free of personal identifiers (as determined by the Health Insurance Portability and Accountability Act—HIPAA) was obtained from three different institutions that encompassed Alzheimer’s Disease Research Centers (ADRCs) at University of California Davis, Columbia University, and University of California San Diego (Fig. 1 – Study Flowchart). Autopsies reflected persons who were denoted to have evaluations for cognitive concerns prior to death and had a recorded pathological diagnosis of AD, of which was defined as having NIA Reagan criteria of intermediate/ high and/or NIA-AA criteria of Intermediate/High AD neuropathologic change [1, 37]. Individuals of two ethnic groups were included: Hispanic and non-Hispanic White decedents, defined based on the participant’s self-reported identification utilizing forms from the National Alzheimer’s Coordinating Center (NACC) [51], genetic determinations were not made. We followed JAMA guidelines on terminology to report race and ethnicity [27]. As this was a retrospective study, data were historical, spanning multiple decades, hence NIA Reagan and NIA-AA criteria were both used.

After evaluating each site, there were 102 decedents who records identified them as having Hispanic ethnicity with available samples. A 2–1 comparison group stratified by site (UCD, UCSD, Columbia) of non-Hispanic White decedents was selected as a random sample from 843 eligible cases. The comparison sample was frequency balanced by sex and by 5-year age group (10 years for the oldest and youngest decades, 50–59 and 100–109, due to small numbers of non-Hispanic White decedents in those groups). Two non-Hispanic White decedents were chosen at random from each cell, or all available in smaller cells. After final definition of the study cohort, the dataset was re-assessed, and cases subsequently found not to meet inclusion criteria were excluded (5 cases reporting an ethnicity other than Hispanic or non-Hispanic White, and 13 cases having no available pathology data) (see Fig. 1 for flowchart). During life, research participants were enrolled in IRB-approved studies at each institution, and at death autopsies were performed after legal consent for autopsy was provided by appropriate family members.

### Clinical comorbidity data

Available information regarding the presence of clinical comorbidities was recorded based on data retrieved from NACC’s Uniform Dataset (UDS) and/or similar forms, which were collected by each institution [51]. Diabetes, hypertension, and/or hyperlipidemia were present if there was history of diagnosis (recorded within the UDS as active and/or inactive) and/or if the participant was mentioned to be taking medication to treat these conditions. Presence of depression was recorded if there was history of diagnosis (active and/or inactive) of depression and/or if the participant was ever-taking antidepressant medication. Presence of stroke and/or trans ischemic attack was recorded if there was mention of history of diagnosis (active and/or inactive).

### Histology and assessments

Brain areas were selected based on availability as well as having consistent sampling across all 3 institutions; here we evaluate the hippocampus, frontal, parietal, and temporal cortices. Prior to processing and assessment, twelve batch numbers were assigned using permuted block randomization within center, gender, and ethnicity stratum. As a measure to minimize potential staining differences across the three sites and due to changes in pathological criteria and antibody staining over time, 5 µm formalin fixed paraffin-embedded (FFPE) sections were cut from the designated anatomic areas available in each institution and stained in the randomized twelve batches at one location (UCD).

All sections were deparaffinized through a graded series of alcohols; unstained slides were placed into two changes of 3 min each into Xylene (HistoPrep™—Fisher Scientific, Pittsburgh, PA, USA), and placed into 2 changes of 100% alcohol (StatLab Medical Products, McKinney, TX, USA) for 2 min each, followed by 2 changes of 95% alcohol for 2 min each. After the deparaffinization was complete, the slides were placed into distilled water. For assessment of Aβ deposits, slides were submitted to pretreatment prior to staining including 10 min in 87% formic acid, endogenous peroxidases were block with 3% Hydrogen Peroxide with subsequent applications of primary and secondary antibodies. The immunohistochemistry staining was performed using the 4G8 monoclonal antibody against Aβ (1:1600; Covance Labs, Madison, WI, USA).

For assessment of tau pathology, the pretreatment used for the AT8 antibody is Heat-Induced-Epitope-Retrieval (HIER). The deparaffinized slides were placed into a plastic coplin jar filled with a Target Retrieval Solution (Citrate Buffer, pH 6.1) and posteriorly placed into a pressure cooker for HIER. Then, slides were stained using a specific antibody for phosphorylated tau, AT8 (1:1000, Thermo Scientific, Waltham, MA, USA). All antibody staining was conducted following standard procedures on automated machines (i.e. autostainers; DAKO AutostainerLink48, Agilent, Santa Clara, CA, USA) utilizing proper positive and negative control for each specific antibody. All staining and immunohistochemistry procedures were performed at the UC Davis Histology Core, a Clinical Laboratory Improvement Amendments (CLIA) and College of American Pathologists (CAP) accredited laboratory operating under the best laboratory practices standards and meets all Federal, State of California, and UC Davis guidelines and regulations.

All immunohistochemistry-stained slides were digitized to obtain whole slide imaging (WSI) using the Zeiss Axio Scan Z.1 scanner at 40 × magnification (0.11 µm/pixel) and files were saved in the proprietary czi format at a 60% compression rate. Semi-quantitative histopathological assessments of each area/stain were conducted by an expert (BD) who was blinded to the demographic, clinical, and genetic information of all cases and their ADRC origin, adapting CERAD and BrainNet Europe semi-quantitative assessments and following guidelines put forth by the NACC Neuropathology form version 10 [4, 14, 15, 48].

The CERAD scoring system was adapted to provide a semi-quantitative assessment of NFTs, cored, diffuse, and neuritic plaques in the densest mm^2^ of tissue area on the slide (none = no pathology denoted as 0, sparse (0–5) denoted as 1, moderate (6–20) denoted as 2, or frequent (greater than 20) denoted as 3); the final score represents the densest area evaluated within the stated subregion for the specific pathology [48].

The Thal amyloid phase scoring system was utilized to denote the anatomic distribution of amyloid plaques in the neocortex, hippocampus, basal ganglia, substantia nigra, and cerebellum, as well as to analyze the neuroanatomical hierarchical course of the disease [72]. The maximum Thal phase was assigned if plaques were observed in: Phase 1: neocortex; Phase 2: hippocampus and entorhinal cortex; Phase 3: putamen; Phase 4: substantia nigra; and Phase 5: cerebellum. Summary of data previously collected by each site are in Table 1.

**Table 1:**
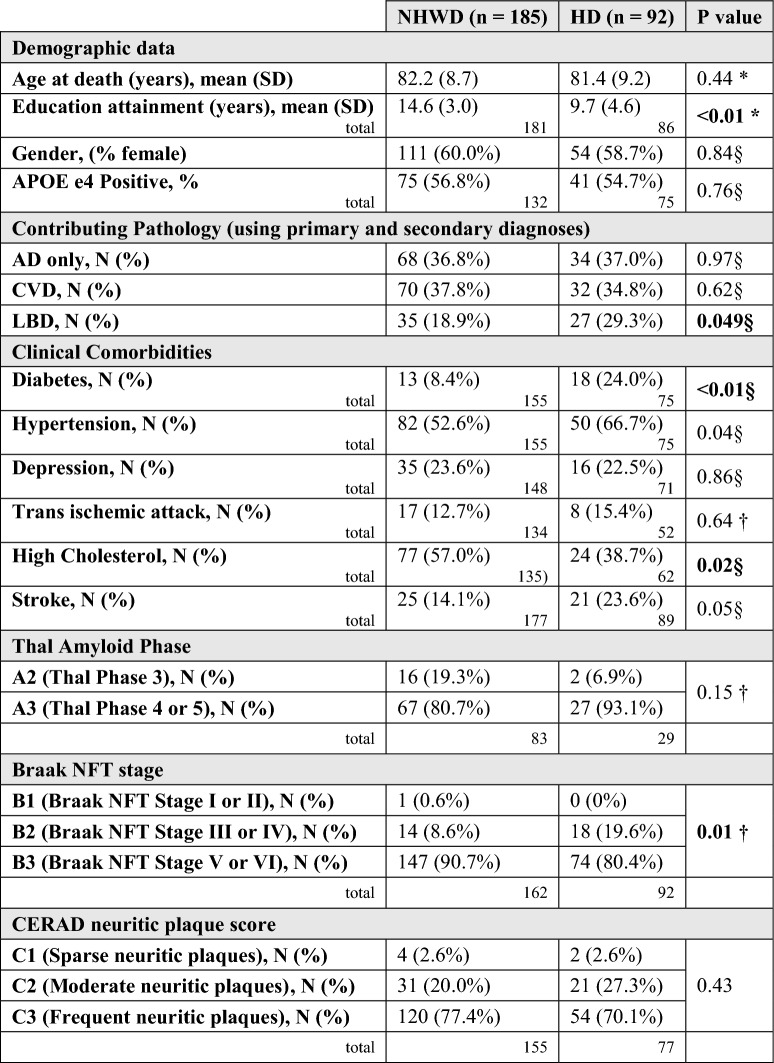
Sociodemographics, select clinical comorbidities, APOE e4 allele positivity, and select pathological data of the study participants, divided by ethnic group (*n* = 277)

Total represents number of cases with available data on the specific variable. All cases had available data on age at death, gender, and primary/secondary pathology diagnoses. Items presented are from historical data collected by each site

AD, Alzheimer’s disease; APOE e4, Apolipoprotein E4; CVD, cerebrovascular dementia; HD, Hispanic decedents; LBD, Lewy Body Disease; NHWD, non-Hispanic White decedents; NFT, neurofibrillary tangle

^§^ Chi-square test

^†^ Fisher exact test

^*^ T-test

Neuropil threads semi-quantitative densities were defined as 0 (0), + (1), +  + (2) or +  +  + (3) according to BrainNET Europe Criteria [4]. Because our study was based on evaluation of tissue and data collected retrospectively, differences in sampling protocols among centers were present. We did not receive and evaluate tissue from the occipital cortex, hence we did not uniformly evaluate Braak NFT stage [15] in these cases. Available data from previous evaluations, albeit done by different experts/neuropathologists over time at the respective centers is included and is present in select Tables to aid with the cohort description.

### Statistical analysis

Demographic, clinical, and neuropathologic characteristics were summarized separately for Hispanic and non-Hispanic White decedents, overall and by site (Table 1). Quantitative variables were summarized by means and standard deviation, and the means for both groups were compared by Student’s two-sample T test. For semi-quantitative or non-normally distributed variables, medians and ranges were provided as summary statistics, and Wilcoxon’s two-sample nonparametric test, using average scores for ties. Analyses of individual variables were restricted to decedents with non-missing data, with no attempt at imputation. To reduce the potential impact of heterogeneity due to the wide range of ages in the sample, we further compared neuropathologic summaries of the two groups by regression analyses, adjusted for age and sex as well as for site (Additional file 1: Table S3). We used linear regression for quantitative variables and ordinal logistic regression for semi-quantitative variables with small numbers of categories. Secondary analyses further compared demographic, clinical, and neuropathologic characteristics across three groups based on Hispanic heritage (Caribbean, Mexican, and Others) and non-Hispanic White decedents, using Kruskal–Wallis nonparametric tests for ordinal categorical variables as an omnibus test of equality across all groups. To assess pairwise differences in neuropathologic characteristics across the four groups, an ordinal logistic regression model was used. The model was adjusted by age and sex, but not site since all Caribbean decedents were from one site. The false discovery rate (FDR) was used for multiple comparisons. All statistical analyses were performed using SAS software (version 9.4, SAS institute, Inc.; Cary, NC, USA). Figures were created using Lucidchart (Lucidchart.com), Biorender (Biorender.com), and R Studio package ggplot2.

## Results

### Demographics

A total of 277 deceased individuals were screened across the three institutes and included in our analyses. As we had a 2:1 matching schema, 33.2% (n = 92) were persons self-identified as Hispanic decedents and 66.8% (n = 185) as Non-Hispanic White decedents. Table 1 summarizes the demographics, neuropathologic, and clinical characteristics of the participants and the groups. Age and gender distributions were nearly identical for Hispanic and non-Hispanic White decedents, reflecting the sampling design (Table 1). The largest Hispanic heritage-based group was of Caribbean heritage (36/92 [39.1%]), predominantly from Puerto Rico (22/92 [23.9%]), followed by Dominican Republic (9/92 [9.8%]), and then Cuba (5/92 [5.4%]). Mexican decedents were the second largest subset of our study cohort (31/92 [33.7%]); fewer Hispanic decedents were of other origins (4/92 [4.3%]), South American descent (3/92 [3.3%]), or of unknown origin (no data available) (18/92 [19.6%]).

Non-Hispanic White decedents averaged five more years of formal education attainment when compared to Hispanic decedents (P < 0.01). The groups were similar in having just over 50% having at least one APOE e4 allele. Some differences were apparent among those with data available on clinical comorbidities, with Hispanic decedents having almost three times higher rates of diabetes (P = 0.01) and two times higher rates of stroke (P = 0.05), but lower proportions of high cholesterol (P = 0.02). Hypertension was present in over half of each group, with higher levels in Hispanic decedents (P = 0.04). Depression was reported in about a quarter of each group, and transient ischemic attack in about 1 in 7 decedents in each group. The proportions with missing data on clinical comorbidities were similar in each group (Table 1).

### Neuropathology

Although all cases had a pathological diagnosis of AD, a primary pathological diagnosis of AD with no secondary pathology present was found in 36.8% of the overall cohort; similar percentages were found for Hispanic (37%) and non-Hispanic White decedents (36.8%) (P = 0.97). The second most frequent diagnosis was cerebral vascular disease (CVD) concomitant with AD, with similar results between groups (Hispanic decedents (34.8%) and non-Hispanic decedents (37.8%) P = 0.62). A third frequent diagnosis of mixed pathologies, Lewy body disease concurrent with AD, was significantly more prevalent among Hispanic individuals (29.3%) than among non-Hispanic White (18.4%) individuals (*P* = 0.04) (Table 1).

**Fig. 2:**
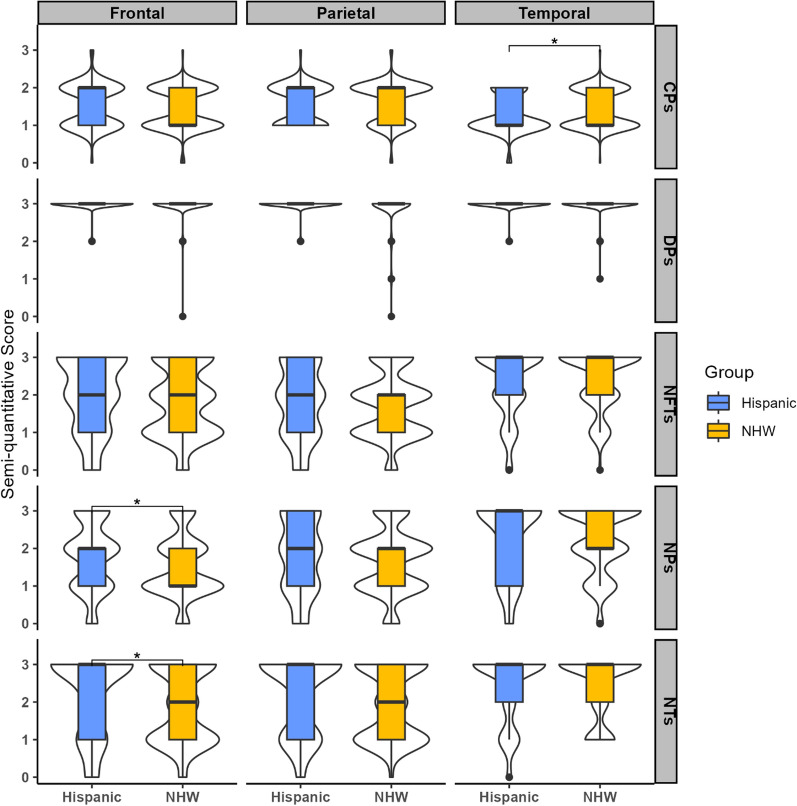
A visualization comprising of a combination of violin and box plots was used to depict the distribution of various pathologies (cored plaques: CPs, diffuse plaques: DPs, neuritic plaques: NPs, neuropil threads: NTs, and neurofibrillary tangles: NFTs) in three specific brain areas-frontal, temporal, and parietal cortices. The violin plots were used to display the distribution of the data, while the boxes indicate the range between the first and third quartiles. The bold horizontal line inside the boxes represents the median value. Additionally, the whiskers extend beyond the upper and lower limits of the box and indicate the range of data within 1.5 times the length of the box

Neuropathologic findings for the posterior hippocampus were similar for both groups, with median levels of 1 (sparse) for core plaques, 2 (moderate) for neuritic plaques, and 3 (frequent) for diffuse plaques, neuropil threads, and NFTs (Table 2). Adjustment via ordinal logistic regression did not alter this finding. Findings in the frontal cortex showed greater levels of neuritic plaques (median 2 vs. 1, *p* = 0.02) and neuropil threads (median 3 vs. 2, *p* = 0.02) for Hispanic decedents than for non-Hispanic White decedents. The groups had similar levels of core plaques, NFTs, and diffuse plaques. Ordinal logistic regression did not modify these findings (Additional File 1: Table S1). In the parietal cortex, levels of core plaques, neuritic plaques, and NFTs were similar in Hispanic and non-Hispanic White decedents with medians of 2, while diffuse plaques had medians of 3 in each group. The median for neuropil threads was 2 in non-Hispanic White and 3 in Hispanic decedents, approaching statistical significance (*p* = 0.06), with some of the difference further accounted for in ordinal logistic regression models (Additional file 1: Table S2). The temporal cortex showed similar findings for both groups for neuritic plaques (medians of 3 for Hispanic decedents and 2 for non-Hispanic White decedents; *p* = 0.56), as well as diffuse plaques, neuropil threads, and NFTs (median of 3 for both groups for all pathologies.) However, even though the median was 1 for both groups, a longer upper tail was observed in the non-Hispanic White decedents for core plaques (*p* = 0.02) (Fig. 2). Additional analyses were carried out adjusting for Braak NFT Stage, Thal phase, and CERAD score, and results did not change substantially,  although there was sparse numbers for lower scores of Braak NFT stage, Thal phase, and CERAD score.

**Table 2:**
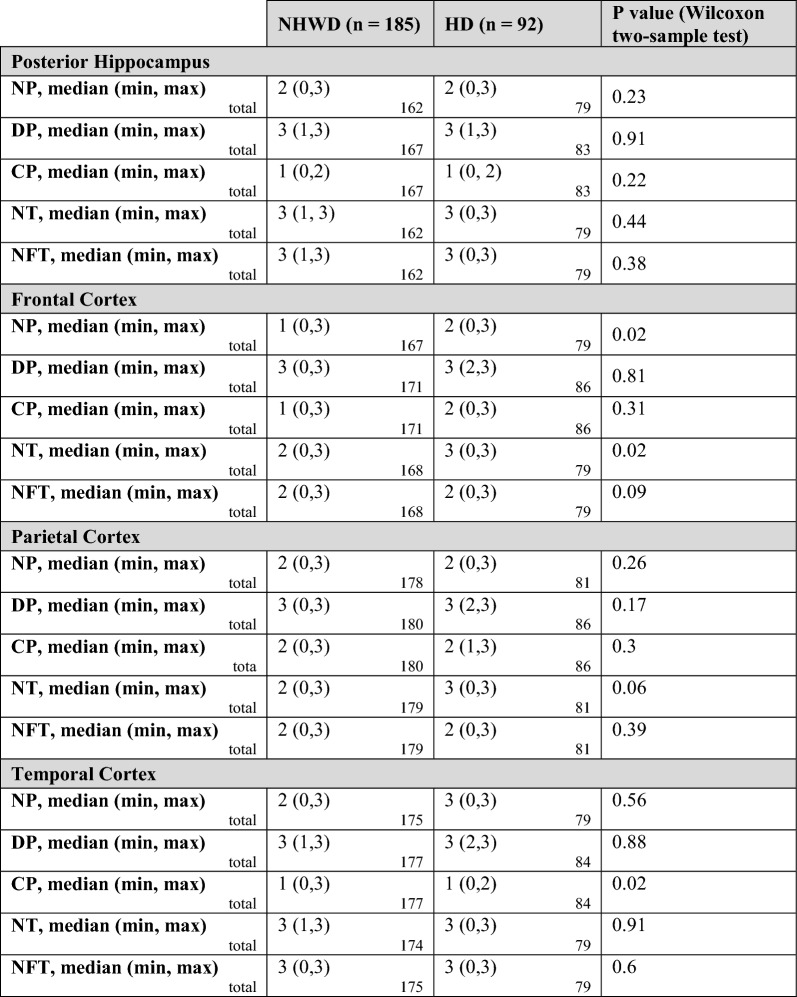
AD-related neuropathological variables in select brain areas, divided by ethnic group (*n* = 277)

Total represents number of cases with available data on the specific variable. Data represent semi-quantitative scores of 0 = none, 1 = mild/sparse, 2 = moderate, 3 = frequent/severe

AD, Alzheimer’s disease; CP, Core plaques; DP, Diffuse plaques; NP, Neuritic plaques; NT, Neuropil threads; NFT, Neurofibrillary tangles

**Fig. 3:**
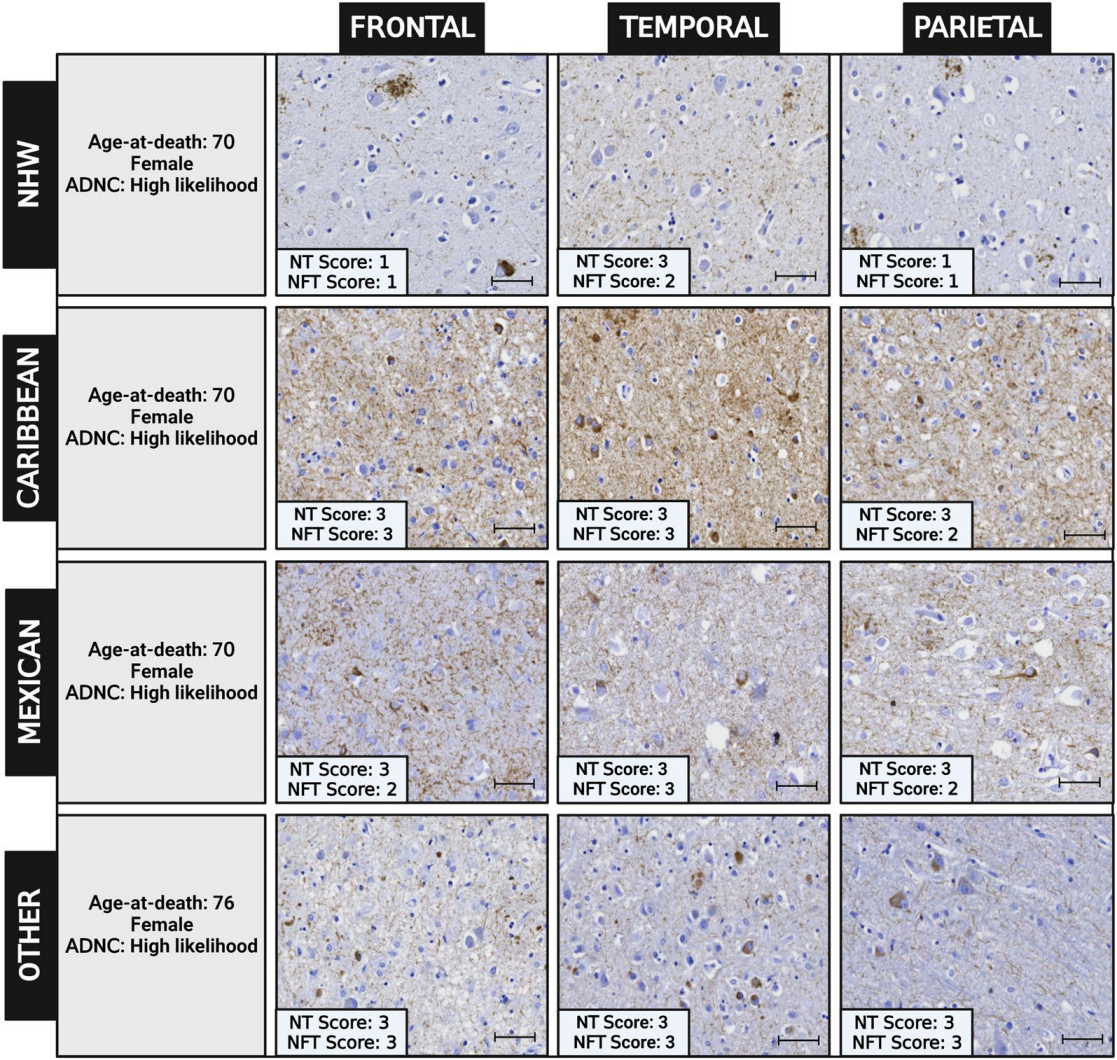
Examples of the histopathologic densities of tau-deposits and corresponding overall regional density scores (for neuropil threads: NTs, and neurofibrillary tangles: NFTs- lower left corner in each image) in three brain regions (frontal, temporal, and parietal cortices). Cases were selected based on heritage group within Hispanic decedents and non-Hispanic White decedents, having similar age at death, gender, and AD likelihood (ADNC = Alzheimer disease neuropathologic change). Scale bar = 50 μm

In secondary exploratory analyses, comparisons further divided the Hispanic participants into three groups based on Hispanic heritage: Caribbean decedents (*N* = 36), Mexican decedents (*N* = 31), and other decedents (*N* = 25) (Additional file 1: Table S2). In the posterior hippocampus, the Caribbean heritage group had higher levels of neuritic plaques and neuropil threads than non-Hispanic White, and the Mexican heritage group (Additional file 1: Tables S2 and S3). Caribbean decedents also had higher levels of neuritic plaques and neuropil threads than Non-Hispanic White decedents in the frontal, parietal, cortices, and NFTs in the frontal cortices (Fig. 3).

## Discussion

We report the neuropathological findings and the frequency distribution of select clinical comorbidities in a research-based autopsy cohort of 277 decedents of Hispanic and non-Hispanic White heritage with AD. Our analysis of semi-quantitative scores using established standardized scales demonstrates Hispanic decedents had greater densities of neuritic plaques and neuropil threads in the frontal cortex, whereas non-Hispanic White decedents had a greater amount of core plaques in the temporal cortex. We also observed a higher range in the score of neuropil threads in the parietal cortex of Hispanic decedents, although it was not significantly different when compared to non-Hispanic White decedents. Interestingly, there were no differences in the pathological findings of the posterior hippocampus between groups. Due to our inclusion criteria and analytic methodology, we had a certain level of homogeneity in terms of age, gender, APOE e4 allele frequency, and final clinicopathological diagnoses (Table 1). There were notable differences in educational attainment and select clinical comorbidities between the groups, with Hispanic decedents having significantly lower educational levels and greater rates of diabetes, hypertension, and stroke, as well as CVD, which are underlying risk factors of AD and may have contributed to the heterogeneous pathological presentation. Our data, provide evidence Hispanic decedents with AD are disproportionately burdened by AD-related pathology, particularly tau deposits, in comparison to non-Hispanic White decedents, based on differences in their pathological profile and severity.

To date, limited neuropathological studies have been conducted to explore disparities in the manifestation of AD in underrepresented populations, particularly in Hispanic persons [26, 31, 57, 64, 69, 76]. In a previous neuropathological study on demented persons, our group reported Hispanic decedents to have a higher incidence of mixed pathologies concurrent with AD, compared to non-Hispanic White decedents who have a significantly higher rate of non-mixed AD (43% vs. 14%) [26]. It is worth noting Hispanic participants in the present study focused on AD also had lower levels of educational attainment and higher rates of co-existing health conditions, which partially align with our previous findings. The lower levels of education in individuals of Hispanic descent might contribute to substantial disparities in their susceptibility to dementia, as it has been shown even a minimal increase in formal education can result in improved cognitive reserve could compensate the neurodegeneration [24, 45]. Interestingly, in the current study we observed a lower percentage of cerebrovascular disease (CVD) in this Hispanic cohort, and this was not different from the non-Hispanic White cohort, in contrast to what we previously reported in the dementia cohort [26]. This discrepancy could be attributed to variations in cohort inclusion/exclusion criteria, the recruitment practices of participants across the three centers, as well as the diversity within the Hispanic cohort. For instance, UC Davis and Columbia include participants regardless of their prior or current cardiovascular risk factors or disease, while UCSD excludes persons having insulin dependent diabetes and major stroke or neurological illness [69] (see Additional file 1: Table S4). There could also be site/temporal differences in the way co-morbidities, such as concomitant pathological diagnoses, are reported. Furthermore, our study included Hispanic individuals from different heritage groups, while prior works focus on select groups [64, 69, 76]. Additional studies delving further into these data are warranted to understand underlying causes of the discrepancies.

Due to the retrospective nature of the study, we utilized the term Hispanic decedents to encompass persons from many different origins; the Hispanic community should not be viewed as one monolithic group [49, 73]. This population has intrinsically diverse genetic, socioeconomic, and cultural characteristics that may help to address the disparities previously reported in AD's clinical presentation [8, 61, 62, 70, 73]. Therefore, to account for this diversity, we performed additional exploratory analyses, although underpowered, to examine potential differences within the Hispanic cohort by creating three groups based on the individuals' self-reported Hispanic heritage: Caribbean, Mexican, and others (including individuals from South America). We also found pathological heterogeneity within Hispanic decedents, with Caribbean decedents having a higher presence of plaques, threads, and NFTs in all four evaluated brain areas, as well as lower levels of education and higher rates of diabetes, hypertension, stroke, and depression compared to the two other Hispanic decedent subgroups, albeit cohort numbers were low having insufficient power and effect size. We also observed in the brains of persons of Mexican descent lower levels of neuropil threads in both the hippocampus and the temporal cortex, even lower than non-Hispanic White decedents (Additional file 1: Table S3). This may aid in explanations of increased AD prevalence in Caribbean individuals [7, 42, 58, 73]. Additionally, with respect to dementia incidence rates Caribbean had higher average frequencies than Mexican persons [10, 25, 58, 71]. Although these results are intriguing, they are underpowered and may well not be representative of the population given the high selectivity of autopsy cohorts. Our data, however, support further study of the role this intrinsic diversity plays in the development of AD [73]. The geographical distribution of the Hispanic population also varies across the United States [2, 22, 57]. Mexican decedents are the largest group and are primarily concentrated in the southwest and south of the country, while Caribbean decedents are more heavily concentrated on the east coast [2, 22]. California and New York are the states with high Hispanic population, ranking first and fourth, respectively [63], which also reflects the origin of our study participants: all evaluated Caribbean participants came from Columbia, and the Mexican participants were mainly from UCSD and UCD.

Generally, the accumulation of tau deposits, in the form of NTs and NFTs, is hypothesized to start in the entorhinal cortex and hippocampus and then spreads to the neocortex. Nonetheless, there are also subtypes of AD with atypical presentation of pathological features, such as sparing the hippocampus [53]. Tau is a microtubule associated protein widely expressed in neurons of the human brain and plays important physiological roles on microtubule assembly and stabilization, as well as promotion of axonal outgrowth [75]. In non-pathological conditions, tau has a naturally unfolded structure, showing a low tendency to aggregation [52]. The correlation between tau pathology and cognitive decline has been well established [20, 32, 43, 55, 56]. Our results did not reveal a significant difference in NFT scoring between groups, although most cases had scores of 2 (moderate) or 3 (frequent) and more finer grain quantitative analyses may aid in providing insight, as has been done with identifying AD subgroups [53]. That stated, our findings did reveal a more pronounced presence of neuropil threads in the frontal and parietal cortices of Hispanic decedents, but no differences within temporal cortices. Typically, neuropil threads are more predominant in comparison to NFTs in immunohistochemically stained sections, which implies that evaluating the presence and density of neuropil threads instead of NFTs may provide more optimal insights into neurodegenerative disease progression, as has been done with the BrainNet Europe Criteria; however, better interrater agreement has been achieved evaluating more severe stages of neuropil threads (Stage V and VI) [4]. To aid in unlocking the secrets of these devastating deposits, more quantitative analysis is needed to aid in understanding potential disease subtypes based on tau pathologies. This variability in severity may arise from several factors including genetic predisposition, concurrent neuropathologies, and/or environmental factors.

Establishing criteria for assessing pathological hallmarks of AD has been a major milestone in the dementia research field, providing a standard procedure for routine diagnostic settings such as NIA Reagan criteria and NIA-AA guidelines (Intermediate or High) [1, 37]. The field has historically used semi-quantitative (CERAD neuritic plaque density) [48] and regional distributions (Thal and Braak) [15, 72] for AD assessment. These disease scales were primarily derived based on the evaluation of the brain tissue of non-Hispanic White individuals, with limited numbers and highly variable methods for sample collection and processing. Specifically, the initial paper on Braak NFT stage was based on a cohort of 83 brains of White individuals of European descent [15]. Furthermore, in our study, we were unable to perform a stage of the tau-related pathology in our study cohort using Braak or BrainNet Europe criteria, as the occipital cortex was not included in the evaluation; available retrospective data collected at each site (UCD, UCSD, Columbia) were included. Further, these criteria employ semi-quantitative scoring methods, which can have interrater variability [5, 33, 36, 47, 54]. Quantitative measurement of pathological features through methods in digital pathology and/or machine learning are a novel and rapidly growing field, particularly promising for providing scalable deeper phenotyping of ADRDs [3, 39, 59, 65, 67, 68, 77]. However, these innovative computer-based evaluation methods require training and validation on large quantities of digitalized and annotated pathology data, which can pose a significant hurdle. To date, many machine learning algorithms have yet to be tested/validated on multiple brain areas to understand generalizability.

Our results should be interpreted with consideration of certain constraints related to data collection and cohort characteristics. Despite applying robust sample selection methods, variation in procedures among centers may contribute to potential bias [26, 31, 74]. The autopsy studies and clinical recruitment within the three institutes are based on a volunteer sample of convenience. Our analyses controlled for the participants' age, gender, and center of origin, providing an increased confidence in our results. However, our results may not be representative of the general Hispanic population affected by AD dementia. Our study utilized case materials collected over a 30-year period. There can be variability in the time intervals between the last clinical evaluation and death, in addition evaluates for specific disorders have evolved over time as well as persons conducting have changed at each of the institutions. Even with careful quality control and data cleaning, there still may be discrepancies, especially within large datasets. This highlights the complexities of retrospective data analyses. Furthermore, the brain tissue evaluated during our study was collected over time, and variations in sampling protocols, preparation methods (e.g., sectioning and/or fixation), and preservation of paraffin-embedded tissue can influence staining quality [13, 29, 66, 74]. To mitigate possible center bias on our results, we centralized slide staining at a single CLIA and CAP certified laboratory with extensive experience conducting pathological staining protocols and choose anatomic areas with similar sample procedures although rostro/caudal medio/lateral and superior/inferior variations can exist. We also only evaluated one 5 µm section per stain and this may not be representative of an entire region. We used randomized block sequences for processing and assessment to further reduce bias. The selected stains (AT8, 4G8) used in this study have previously been used within numerous centers and in published scales [74]. Hence given these limitations, we focused analyses on the neuropathology variables collected in a standardized fashion through this study and to highlight the heterogeneity of the pathological changes.

Despite the limitations, this study has numerous strengths. To our knowledge, this is the largest autopsy-based study focused on Hispanic decedents with AD. We have measured the hallmark pathologies of AD using validated semi-quantitative measurements, providing an unbiased evaluation across multiple brain regions. The participants of the two groups had systematic pathological assessments performed by a single individual blind to all information pertaining to the cases, reducing potential interrater variability and evaluation bias. This innovative multi-center autopsy-based study presents a foundation of novel information about the neuropathological landscape heterogeneity of AD among a cohort of Hispanic and non-Hispanic White decedents matched on demographic features.

In summary, our results indicate adjusting for age, sex, and center of origin, Hispanic decedents with a pathological diagnosis of AD prior to death have greater levels of tau pathology in select brain regions when compared to non-Hispanic White decedents, demonstrating differences of the neuroanatomical distribution and severity of AD-related pathology. Although the focus of this work was to characterize the differences in the hallmark pathology of AD, further research is needed to elucidate the role of other common dementia-related pathologies (such as CVD and Lewy body disease) and how their interaction influences disease onset and progression in persons of Hispanic descent. It is vital to note these historic categories are social constructs and cultural and social associations may underlie differences. Additional studies using innovative quantitative methods can aid in broadening these findings, providing detailed brain tissue phenotyping in more ethnically diverse groups. Overall, the results we present here emphasize the importance of more thorough deeper phenotyping of the AD neuropathological landscape among diverse ethnic cohorts for enhanced clinical correlations and precision medicine advancement [67]. We hope to contribute to filling the historical knowledge gap about how ethnicity may interplay with genetic, sociocultural, and environmental factors affecting the prevalence and trajectory of ADRDs among underrepresented populations, ultimately aiming for better preventive measures, treatments, and prognoses for all individuals.

## Supplementary Information

**Additional file 1.** Supplementary Tables

## Data Availability

If accepted for publication and after journal release of the formal formatted manuscript, associated data will be available through Dryad (https://datadryad.org/stash). Dryad, is a tool for researchers to describe, upload, and share their research data. Datasets published in Dryad receive a citation and can be versioned at any time.
