# Supplementary material for: The neuropathological landscape of Hispanic and non-Hispanic White decedents with Alzheimer disease
Source: Acta Neuropathol Commun. 2023 Jun 29;11:105. doi: 10.1186/s40478-023-01574-1 (PMC10311731; doi:10.1186/s40478-023-01574-1)
Supplement: Supplementary file 1 — Additional file 1. Supplementary Tables [file 40478_2023_1574_MOESM1_ESM.docx]

**Supplementary Tables**

**Supplementary Table 1.** Regression analysis. Based on the Ordinal Logit Model 5, Hispanic decedents (HD) have higher odds of having higher score of neuritic plaques (1.76 times) and neuropil threads (2.14 times) in frontal cortex than non-Hispanic white decedents (NHWD). On the other hand, HD have 50% lower odds of having high score of core plaques in temporal cortex than NHWD.

| **Ordinal Logit Model5** | **Neuritic Plaques (frontal)** | | | **Neuropil Threads (frontal)** | | **Core Plaques (temporal)** | |
| --- | --- | --- | --- | --- | --- | --- | --- |
|  | Odds Ratio (95% CI) | P value | Odds Ratio (95% CI) | | P value | Odds Ratio (95% CI) | P value |
| Race = Hispanic | 1.76 (1.05, 2.96) | 0.03 | 2.14 (1.18, 3.89) | | 0.01 | 0.5 (0.28, 0.89) | 0.02 |
| Gender = Female | 1.15 (0.66, 1.98) | 0.62 | 1.28 (0.7, 2.32) | | 0.42 | 0.89 (0.50, 1.59) | 0.69 |
| Age at death (years) | 0.93 (0.90, 0.97) | **<0.01** | 0.93 (0.89, 0.96) | | **<0.01** | 1.01 (0.98, 1.05) | 0.38 |
| Columbia | 4.25 (2.27, 7.95) | **<0.01** | 17.78 (8.6, 36.68) | | **<0.01** | 0.99 (0.53, 1.84) | 0.2 |
| UC Davis | 3.72 (1.84, 7.53) |  | 6.97 (3.24, 15.0) | |  | 0.52 (0.24, 1.12) |  |

References of:

race = NHWD,

sex = male,

center = UCSD,

Ordinal Logit Model5: pathology score (0, 1, 2, 3) = race (HD vs NHWD) + sex + death age + center + random (batch)

**Supplementary Table 2.** Pattern of AD-related neuropathological variables in select brain areas, divided by ethnic group, with the Hispanic decent group subdivided based on Hispanic heritage (*n* = 277)

|  | **NHWD**  **(n = 185)** | **Caribbeans (n = 36)** | **Mexicans (n = 31)** | **Others (n = 25)** | **P value (Kruskal Wallis test)** |
| --- | --- | --- | --- | --- | --- |
| **Posterior Hippocampus** | | | | | |
| **NP, median (min, max)**  total | 2 (0,3)  162 | 2 (1,3)  33 | 1.5 (0,3)  26 | 2 (0,3)  20 | **<0.01** |
| **DP, median (min, max)**  total | 3 (1,3)  167 | 3 (1,3)  32 | 3 (1,3)  28 | 3 (1,3)  23 | 0.2 |
| **CP, median (min, max)**  total | 1 (0,2)  167 | 1 (0,2)  32 | 1 (0,2)  28 | 1 (0,2)  23 | 0.58 |
| **NT, median (min, max)**  total | 3 (1, 3)  162 | 3 (2,3)  33 | 1.5 (0,3)  26 | 3 (0,3)  20 | **<0.01** |
| **NFT, median (min, max)**  total | 3 (1,3)  162 | 3 (1,3)  33 | 3 (1,3)  26 | 2.5 (0,3)  20 | 0.26 |
| **Frontal cortex** | | | | | |
| **NP, median (min, max)**  total | 1 (0,3)  167 | 2 (1,3)  30 | 1.5 (0,3)  28 | 2 (0,3)  21 | **<0.01** |
| **DP, median (min, max)**  total | 3 (0,3)  171 | 3 (2,3)  31 | 3 (2,3)  31 | 3 (3,3)  24 | 0.67 |
| **CP, median (min, max)**  total | 1 (0,3)  171 | 1 (1,3)  31 | 2 (1,3)  31 | 2 (0,2)  24 | 0.67 |
| **NT, median (min, max)**  total | 2 (0,3)  168 | 3 (1,3)  30 | 2 (0,3)  28 | 1 (0,3)  21 | **<0.01** |
| **NFT, median (min, max)**  total | 2 (0,3)  168 | 3 (1,3)  30 | 2 (0,3)  28 | 1 (0,3)  21 | **<0.01** |
| **Parietal cortex** | | | | | |
| **NP, median (min, max)**  total | 2 (0,3)178 | 2 (1,3)  33 | 1 (0,3)  28 | 1.5 (0,3)  20 | **<0.01** |
| **DP, median (min, max)**  total | 3 (0,3)  180 | 3 (2,3)  34 | 3 (3,3)  29 | 3 (3,3)  23 | 0.22 |
| **CP, median (min, max)**  total | 2 (0,3)  180 | 2 (1,2)  34 | 1 (1,2)  29 | 2 (1,3)  23 | 0.29 |
| **NT, median (min, max)**  total | 2 (0,3)  179 | 3 (1,3)  33 | 1 (0,3)  28 | 3 (0,3)  20 | **<0.01** |
| **NFT, median (min, max)**  total | 2 (0,3)  179 | 2 (1,3)33 | 1.5 (0,3)  28 | 2 (0,3)  20 | 0.12 |
| **Temporal cortex** | | | | | |
| **NP, median (min, max)**  total | 2 (0,3)  175 | 3 (1,3)  33 | 2 (0,3)  27 | 2 (0,3)  19 | **<0.01** |
| **DP, median (min, max)**  total | 3 (1,3)  177 | 3 (2,3)  33 | 3 (1,3)  29 | 3 (3,3)  22 | 0.65 |
| **CP, median (min, max)**  total | 1 (0,3)  177 | 1 (1,2)  33 | 1 (0,2)  29 | 1 (0,2)  22 | 0.07 |
| **NT, median (min, max)**  total | 3 (1,3)  174 | 3 (1,3)  33 | 2 (0,3)  27 | 3 (0,3)  19 | **<0.01** |
| **NFT, median (min, max)**  total | 3 (0,3)  174 | 3 (1,3)  33 | 2 (0,3)  27 | 3 (0,3)  19 | **0.01** |

AD, Alzheimer’s disease; CP, Core Plaques; DP, Diffuse Plaques; NP, Neuritic Plaques; NT, Neuropil Threads; NFT, neurofibrillary tangles; NHWD, non-Hispanic White decedents

*** Caribbean heritage group: (N=5 Cuban, N=9 Dominican, N=22 Puerto Rican): all from Columbia University)**

*** Other heritage group: (N=18 Unknown, N=4 Others, N=3 South American) (n=7 Columbia University, n=10 UCD, n=8 UCSD)**

*** Mexican heritage group: (N=1 Columbia University, N=7 UCD, N=23 UCSD**

**Supplementary Table 3. OR (95%CI) from pairwise comparison of heritage groups, using ordinal logistic regression, adjusted for age and sex and corrected for multiple comparison via False Discovery Rate (FDR).**

*** indicates P<0.05 after multiple comparisons adjustments**

|  | **Mexican vs NHW** | **Caribbean vs NHW** | **Others vs NHW** | **Mexican vs Others** | **Caribbean vs Others** | **Caribbean vs Mexican** |
| --- | --- | --- | --- | --- | --- | --- |
| **PHippo NP** | 0.62 (0.26, 1.45) | **2.75 (1.31, 5.77) *** | 1.11 (0.45, 2.72) | 0.56 (0.17, 1.80) | 2.48 (0.83, 7.42) | **4.44 (1.53, 12.86) *** |
| **PHippo NT** | 0.40(0.17, 0.96) | **5.58 (1.59, 19.62) *** | 1.11 (0.43, 2.89) | 0.36 (0.11, 1,22) | 5.00(1.10, 22.84) | 14.01 (3.20, 61.39) |
| **MFG NP** | 1.32 (0.61, 2.86) | **3.55 (1.70, 7.43) *** | 1.09 (0.45, 2.64) | 1.22 (0.40, 3.66) | 3.26 (1.11, 9.57) | 2.68 (1.01, 7.15) |
| **MFG NT** | 1.01 (0.46, 2.21) | **14.34 (4.04, 50.84) *** | 0.76 (0.30, 1.93) | 1.33 (0.42, 4.22) | **18.90 (4.11, 86.92) *** | **14.18 (3.41, 58.93) *** |
| **MFG NFT** | 0.96 (0.45, 2.03) | **5.47 (2.38, 12.53) *** | 0.59 (0.23, 1.51) | 1.62 (0.52, 5.04) | **9.26 (2.79, 30.69) *** | **5.72 (2.02, 16.24) *** |
| **Pari NP** | 0.64 (0.30, 1.37) | **3.24 (1.54, 6.83) *** | 0.63 (0.25, 1.57) | 1.02 (0.33, 3,17) | **5.18 (1.68, 15.95) *** | **5.08 (1.86, 13.90) *** |
| **Pari NT** | 0.76 (0.35, 1.68) | **4.08 (1.64, 10.17) *** | 1.28 (0.47, 3.46) | 0.60 (0.18, 2.01) | 3.20 (0.88, 11.60) | **5.35 (1.70, 16.76) *** |
| **Temp NT** | **0.37 (0.16, 0.84) *** | **4.65 (1.32, 16.32) *** | 0.76 (0.29, 2.00) | 0.49 (0.15, 1.62) | **6.13 (1.34, 28.11) *** | **12.56 (2.99, 52,80) *** |
| **Temp NFT** | **0.32 (0.14, 0.71) *** | 1.28 (0.56, 2.96) | 0.99 (0.37, 2.61) | 0.32 (0.10, 1.05) | 1.30 (0.39, 4.34) | **4.06 (1.38, 11.93) *** |

NHW, non-Hispanic White group; NP, Neuritic Plaques; NT, Neuropil Threads; NFT, neurofibrillary tangles; P Hippo, posterior hippocampus; MFG, middle frontal gyrus; Pari, parietal; Temp, temporal

**Supplementary Table 4.** Center differences

|  | **Columbia (N=132)** | **UC Davis (N=55)** | **UCSD (N=90)** | **P value** |
| --- | --- | --- | --- | --- |
| **Demographic** | | | | |
| **Age at death (years), mean (SD)** | 79.2 (9.6) | 84.9 (7.4) | 84.2 (7.1) | **<0.01 *** |
| **Education, mean (SD)**  total | 13.0 (4.5)  (122) | 12.4 (3.9)  (55) | 13.5 (4.3)  (90) | 0.3 * |
| **Gender, N (% female)** | 93 (70.5%) | 27 (49.1%) | 45 (50%) | **<0.01 §** |
| **APOE e4 Positive, N (%)**  total | 45 (58.4%)  (77) | 26 (65%)  (40) | 45 (50%)  (90) | 0.24 § |
| **Contributing Pathology (using primary and secondary diagnoses)** | | | | |
| **AD only, N (%)** | 36 (27.3%) | 24 (43.6%) | 42 (46.7%) | **< 0.01 §** |
| **CVD, N (%)** | 78 (59.1%) | 15 (27.3%) | 9 (10%) | **< 0.01 §** |
| **LBD, N (%)** | 24 (18.2%) | 14 (25.5%) | 24 (26.7%) | 0.27 § |
| **Clinical comorbidities** |  |  |  |  |
| **Diabetes, N (%)**  total | 17 (19.8)  86 | 5 (9.3%)  54 | 9 (10%)  90 | 0.12 † |
| **Hypertension, N (%)**  total | 50 (57.5%)  87 | 28 (51.9)  54 | 54 (60%)  90 | 0.63 § |
| **High Cholesterol, N (%)**  total | 35 (40.2%)  87 | 14 (46.7%)  30 | 52 (65%)  80 | **<0.01 §** |
| **Stroke, N (%)**  total | 25 (20.7%)  121 | 13 (23.6%)  55 | 8 (8.9%)  90 | 0.03 §  0.02 † |
| **Trans ischemic attack, N (%)**  total | 8 (15.1%)  53 | 5 (9.3%)  54 | 12 (15.2%)  79 | 0.58 † |
| **Depression, N (%)**  total | 44 (36.4%)  121 | 5 (27.8%)  18 | 2 (2.5%)  80 | **<0.01 †** |
| **Thal Amyloid Phase** | | | | |
| **A2 (Thal Phase 3), N (%)** | 18 (27.3%) | 0 (0%) | 0 (0%) | **<0.01 †** |
| **A3 (Thal Phase 4 or 5), N (%)** | 48 (72.7%) | 6 (100%) | 40 (100%) |  |
| total | 66 | 6 | 40 |  |
| **Braak NFT stage** | | | | |
| **B1 (Braak Stage I or II), N (%)** | 1 (0.8%) | 1 (2.3%) | 0 (0%) | **<0.01 †** |
| **B2 (Braak stage III or IV), N (%)** | 4 (3.3%) | 12 (27.3%) | 15 (16.7%) |  |
| **B3 (Braak stage V or VI), N (%)** | 115 (95.8%) | 31 (70.4%) | 75 (83.3%) |  |
| total | 120 | 44 | 90 |  |
| **CERAD neuritic plaque score** | | | | |
| **C1 (Sparse neuritic plaques), N (%)** | 4 (3.7%) | 0 | 2 (2.2%) | **<0.01 †** |
| **C2 (Moderate neuritic plaques), N (%)** | 6 (5.5%) | 12 (36.4%) | 34 (37.8%) |  |
| **C3 (Frequent neurtic plaques), N (%)** | 99 (90.8%) | 21 (63.6%) | 54 (60%) |  |
| total | 109 | 33 | 90 |  |

AD, Alzheimer’s disease; APOE e4, Apolipoprotein E4; CVD, cerebrovascular dementia; HD, Hispanic decedents; LBD, Lewy Body Disease; NHWD, non-Hispanic White decedents

§ Chi-square test

† Fisher exact test

* ANOVA test
